# COMPARISON OF CONVENTIONAL HAND EXAMINATION ON SIX OPTIMISED DR SYSTEMS

**DOI:** 10.1093/rpd/ncab067

**Published:** 2021-05-10

**Authors:** Helle Precht, Claus Bjørn Outzen, Martin Weber Kusk, Malene Bisgaard, Dag Waaler

**Affiliations:** Health Sciences Research Centre, UCL University College, Niels Bohrs Allé 1, 5230 Odense M, Denmark; Department of Radiology, Kolding, Lillebaelt Hospital, University Hospitals of Southern Denmark, Sygehusvej 24, 6000 Kolding, Denmark; Department of Regional Health Research, University of Southern Denmark, J.B. Winsløws Vej 19, 3., 5000 Odense C, Denmark; Health Sciences Research Centre, UCL University College, Niels Bohrs Allé 1, 5230 Odense M, Denmark; Department of Radiology and Nuclear Medicine, University Hospital of Southwest Denmark, Finsensgade 35, 6700 Esbjerg, Denmark; Health Sciences Research Centre, UCL University College, Niels Bohrs Allé 1, 5230 Odense M, Denmark; Department of Health Sciences, Gjøvik, Norwegian University of Science and Technology, Teknologiveien 22, 2803 Gjøvik, Norway

## Abstract

The purpose of this study was to investigate the challenges in comparing digital radiography (DR) systems from different vendors for various combinations of exposure factors in posterior–anterior hand radiographs. Image quality was evaluated for a range of tube voltages and tube current-time products using a technical contrast-detail (CDRAD) phantom and an anthropomorphic hand phantom. 900 technical CDRAD images were analysed providing quality figures of merit (IQF_inv_) and two experienced reporting radiographers using visual grading analysis (VGA) scored 108 anthropomorphic images. This study demonstrates the differences between the DR systems included. When compensating for variations in dose, Canon showed superior results for technical image quality and Fuji for visual image quality for a standard dose point at DR hand examination (ln(DAP) 1.1, 50 kV and 2.5 mAs).

## INTRODUCTION

Digital radiography (DR) examinations are among the most dominating procedures in medical imaging departments ^(1)^. DR systems from different vendors each have their own unique combination of X-ray generation and detection hardware, as well as software for image post processing. Choice of system is often a compromise between clinical demands, economy and personal preferences. Thus, producing uniform image quality in departments with different DR systems, while adhering to ALARA principles can be challenging ^(2–4)^. When switching from one system to another, a simple method to compare clinical image quality related to dose would be of great help when setting up exposure parameters. Using a technical phantom for this procedure, would simplify the process, while eliminating confounding factors from variations in patients’ anatomy and size ^(5,6)^.

The CDRAD contrast-detail phantom is a method designed to evaluate technical image quality, providing a simple figure of merit (IQF_inv_), which can be calculated automatically using dedicated software ^(7,8)^. Within the same DR system, significant correlation between IQF_inv_ and clinical image quality observed on anthropomorphic phantoms and human cadavers has been described ^(9–11)^. Similarly studies of chest and pelvis radiography have shown significant correlation between various technical image quality indicators and subjective, clinical image quality ^(2,12)^. If this could be expanded to compare clinical image quality from different DR systems, it would be of great help.

The purpose of this study was to compare DR systems from different vendors for various combinations of exposure factors in posterior–anterior hand radiographs, using both technical and visual image quality parameters.

## MATERIALS AND METHODS

Six DR vendors were invited to this project, all collaborating around system specifications and optimisation of the system. Each vendor pointed out a specific hospital, X-ray room and DR system they wanted to include in the project. Each of them optimised their software parameter combinations to achieve optimal image quality for hand examinations used for the experimental exposures.

### The DR systems

Image quality related to dose was compared from the optimised DR systems. The six DR systems were Agfa, Canon, Fuji, GE, Philips and Siemens. See system specifications in Table 1.

**Table 1:** Specifications on the six DR systems.

	Detector	Detector area (cm × cm)	Image matrix	Pixel pitch (μm)	Scintillator type	DQE	A/D (Grayscale) bit depth	Permanent beam filtration (mmAl)
Agfa	CXDI-70C	35 **×** 43	2800 **×** 3408	125	CsI/a-Si	>60%@0 lp/mm	16 (12)	3.6
Canon	CXDI-80C	42 **×** 42	3360 **×** 3360	125	CsI/a-Si	>60%@0 lp/mm	14 (12)	2.5
Fuji	FDR D-evo plus C35s	46 **×** 38	2880 **×** 2304	160	CsI/a-Si	65%@0 lp/mm 40%@2 lp/mm	16	3.0
GE	Definium 6000	43 **×** 43	2048 **×** 2048	210	CsI/a-Si	74%@0 lp/mm	14	2.8
Philips	Trixell Pixium 4600	43 **×** 43	3001 **×** 3001	143	CsI/a-Si	65%@0.05 lp/mm	14	2.5
Siemens	Trixell Pixium FE3543pR	35 **×** 43	2364 **×** 3001	143	CsI/a-Si	70%@0.01 lp/mm	16	2.5

### Exposure parameter settings

The exposure parameters were chosen as closely to common clinical practice as possible. These were set the same way for all DR systems, with some few slight exceptions (explained in bracket), based on limitations in the current system. Tube voltage (kVp) was set at 45 (46 kVp for Philips), 50 and 55. For each tube voltage level, ranges of tube current-time products (mAs) were used as: 0.5, 0.63 (only used for the CDRAD experiments, 0.60 for Canon, Fuji and Philips and 0.64 mAs for Agfa), 0.8, 1.0 (only CDRAD), 1.25, 1.6 (only CDRAD), 2.5 and 3.2 (3.1 mAs for Philips). The source-image-distance was fixed at 100 cm, only small focal spot was used and the collimation field sizes were 17*29.5 cm^2^ (VGA) and 26.5*26.5 cm^2^ (CDRAD). All vendors gave their inputs and accepted the given technical parameters before the experiments started.

Dose-area-product (DAP) values were recorded for all exposures. Although the systems used (almost) similar tube voltage and tube-current-time product values, the vendors used slightly different X-ray filtrations see Table 1. Such differences might be important when comparing system quality performances. DAP values were measured using each system’s built-in DAP-meter, and although these are subject to periodic calibration, there may be systematic deviations between them causing bias in the comparisons. To correct for this, a common skin dose meter (PSD—patient skin dosimeter, Unfors, Stockholm, Sweden) was used as calibration between the systems. For each system, 10 repeated skin doses and DAP measurements, at exposure 1.6 mAs and for each of the tube voltage settings (45, 50 and 55), were made. The average skin-dose-to-DAP-ratios were then used to calibrate the recorded DAP values between the systems.

### Technical image quality analysis

To assess reliability of dose measurements and software processing parameters, all exposures for the technical quality assessments were repeated 10 times at each tube voltage and tube-current-time combination. The technical quality of the 900 images (15 images repeated 10 times on six DR systems) were assessed using the CDRAD 2.0 phantom from Artinis ^(7,8)^.

An ‘inverse image quality figure’ (IQF_inv_) was calculated from the equation ${\mathrm{IQF}}_{\mathrm{inv}}=\frac{100}{\sum_{i=1}^{15}{C}_i{D}_{i,\mathrm{th}}}$, where *D*_*i*,th_ represents the smallest detectable diameter (threshold diameter, in mm) of the hole in column *i*, and *C_i_* represents the depth (in mm) of that hole.

The images were analysed by the Analyser 2.0 software, using default input parameters ^(7,8)^. To investigate and compare quality performances between the systems, two regression models were analysed, (1) a power function model (IQF_inv_ *= α·*DAP*^β^*) and (2) a logarithmic model (IQF_inv_ *= a + b·*ln(DAP)). The IQF_inv_ and DAP values used in the regressions were averages of 10 repeated exposures with the same exposure settings.

### Visual image quality analysis

Visual evaluation of the anthropomorphic hand images was performed using absolute visual grading analysis (VGA)^(9,10)^. For each of the exposure settings, 10 exposures were made, from which the image with the median DAP reading was chosen for visual evaluation. Thus, 108 images (15 images for each of the six DR system and three repeated images for each system for intra-observer agreement assessments) were scored by two experienced radiographers with a postgraduate degree in reporting of the appendicular/axial skeleton. The VGA score used predefined image criteria inspired by European Guidelines ^(13)^, developed in collaboration with the observers. These were individually scored on a scale from 1 to 5 (Table 2).

**Table 2:** VGA image criteria and VGA scale for scoring image quality^(11,13,14)^, and relation to the technical quality parameters spatial resolution, contrast and noise^(9,12,15)^.

No	VGA image criteria	No	Relation to technical image quality
1	Visually sharp demarcation between cancellous and compact bone in the proximal phalanx	1	Spatial resolution at low contrast
2	Visually sharp reproduction of trabecular structures proximally in third fingers middle phalanx	2	Spatial resolution at high contrast
3	Homogeneous reproduction of the adjacent soft tissues	3	Noise
4	Visually sharp demarcation of third fingers metacarpophalangeal joint	4	Contrast for medium structures
5	Visually sharp demarcation of third fingers proximal interphalangeal joint	5	Contrast for small structures

The anthropomorphic images were analysed by the observers in a randomised order and blinded to both vendor and exposure settings using ViewDEX (Viewer for Digital Evaluation of X-ray images)^(11,14)^. The ratings of each observer were recorded directly on the computer. To reduce bias in the VGA scores, the observers were given unlimited time to work undisturbed at the same location, using the same calibrated diagnostic monitor (EIZO RX850, Hakusan, Japan) and physical surroundings. The evaluation took place in a hospital at the regular image reading workstations. To reduce inter-observer variation, a training session was performed prior to the image evaluation, to illustrate best and worst possible image qualities. The scorings from the training session were not used in the analysis.

The results of the VGA study was summarised in an overall quality score using the equation $\mathrm{VGAS}=\frac{\sum_{O,I}{S}_c}{NiNo}$, where *S_c_* is the individual score for observer *O* (reporting radiographer) and image criterion *I*, *N_i_* is the total number of image criteria and *N_O_* the total number of observers ^(10)^.

The relationship between visual image quality and recorded dose (DAP) was analysed using the same regression models as for the technical image quality, i.e. (1) a power function model (VGAs *= α·*DAP*^β^*) and (2) a logarithmic model (VGAs *= a + b·*ln(DAP)).

Inter- and intra-observer-agreements were evaluated using Cohens weighted kappa.

All statistical analysis was performed using Excel (Microsoft Corp., WA) and STATA 13 (StataCorp, TX).

## RESULTS

### DAP analysis

The DAP calibration analysis between the systems, using the standard skin-dose meter, yielded calibration factors ranging from 0.93 (Philips); 0.99 (Agfa and Fuji); 1.01 (Siemens) and 1.03 (GE) to 1.05 (Canon). These factors were multiplied with the measured DAP values to get calibrated and comparable values.

**Figure 1:**
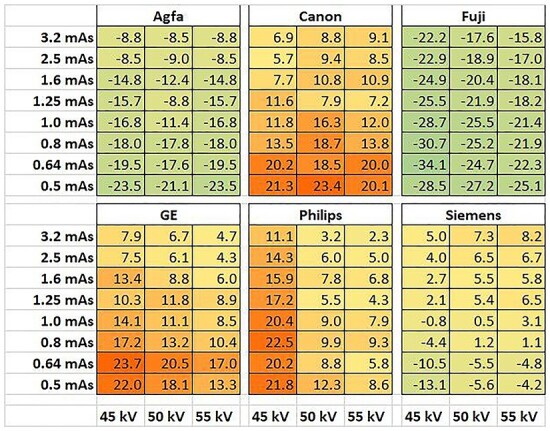
Calibrated DAP variations from the CDRAD study, presented as percentage differences between DAP-values for each system and the average DAP value across all systems for each exposure parameter combination. The scale ranges from the lowest observed difference from the average (green) to the highest (red).

A heat map of each system’s variations in calibrated DAP values for all exposure combinations in the CDRAD experiments, presented as deviation in % from the average DAP value across all systems is shown in Figure 1. Uncertainties (95% confidence intervals) in DAP-measurements were estimated from the 10 repeated measurements to be in the range 2.6%, for 0.5 mAs, to 0.6% for 3.2 mAs. As visualised in Figure 1, there are important differences between the systems, and these need to be considered when comparing the system’s quality performances. Similar results were obtained for the VGAs experiments.

The DAP deviations between the systems, shown in Figure 1, correspond well (although not statistically significant, *p* = 0.13) with the amount of X-ray beam filtration see Table 1. The elevated values for Philips at 45 kV can probably be attributed to the fact that the actual tube voltage value for the Philips system was 46 kV.

### DAP adjusted image quality comparisons

The power model regression results for IQF_inv_ and VGAs, respectively, vs. DAP are shown in Table 3.

**Table 3:** Results of power function modelling of i) IQF_inv_ *= α·*DAP*^β^* and ii) VGAs *= α·*DAP*^β^,* respectively.

	i) IQF_inv_ *= α·*DAP^β^			ii) VGAs *= α·*DAP^β^		
			*R^2^*			*R^2^*
Agfa	3.03 (2.84–3.21)	0.67 (0,59–0.74)	0.94	2.98 (2.82–3.16)	0.34 (0.28–0.40)	0.91
Canon	4.58 (4.39–4.77)	0.36 (0.31–0.42)	0.90	3.71 (3.57–3.85)	0.07 (0.01–0.12)	0.37
Fuji	2.64 (2.54–2.76)	0.60 (0.54–0.66)	0.96	3.42 (3.16–3.69)	0.34 (0.25–0.44)	0.83
GE	3.00 (2.90–3.10)	0.44 (0.40–0.48)	0.95	3.44 (3.23–3.65)	0.23 (0.15–0.31)	0.73
Philips	2.56 (2.48–2.64)	0.31 (0.27–0.35)	0.93	2.67 (2.51–2.84)	0.39 (0.32–0.47)	0.92
Siemens	4.03 (3.94–4.22)	0.44 (0.40–0.48)	0.96	3.72 (3.59–3.89)	0.16 (0.12–0.22)	0.81
Average	3.28 (3.18–3.39)	0.47 (0.42–0.51)	0.96	3.34 (3.22–3.46)	0.24 (0.20–0.29)	0.91

*Note*: *R^2^* is the squared Pearson correlation coefficient for the fit.

### Technical image quality

**Figure 2:**
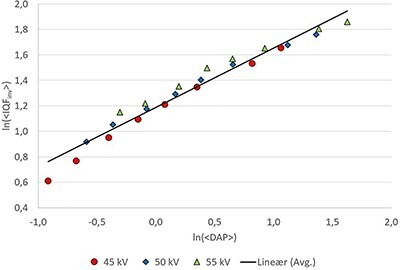
ln(< IQF_inv_>) vs. ln(<DAP>) (where < > symbolises averages from 10 repeated exposures) for all exposure parameter combinations averaged over all systems. In this plot, the slope corresponds to the β-exponent in the power regression model. Note that the reported value for the squared Pearson correlation coefficient (*R*^2^) is for the averaged data over all three tube voltages

A plot of the averaged data points across all systems for the CDRAD experiments (IQF_inv_ *= α·*DAP*^β^*) is presented in Figure 2.

### Technical and visual image quality evaluation

The results of the logarithmic data modelling, VGAs *=* a *+ b·*ln*(*DAP*)* and IQF_inv_ *= a + b·*ln*(*DAP*)*, respectively, are shown in Table 4.

**Figure 3:**
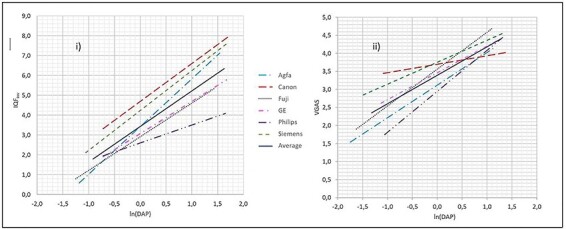
Linear regression results of IQF_inv_ (**a**) and VGAS (**b**), respectively, vs. ln(DAP), highlighting the differences in technical and visual quality figures of merit between the DR systems. The individual data points are omitted to make it easier to visually compare the systems. Regression results are shown in Table 4.

The logarithmic regression results are illustrated in Figure 3, showing pronounced differences in DAP-sensitivities between both the technical and visual quality assessments for each DR system, as well as between them.

### Inter- and intra-observer agreements

Relatively good intra-observer agreement for both radiographers was found with Kappa values varying from 0.71 to 0.93 for most image criteria, except VGA criteria 4 and 5 for observer B. To compare inter-observer agreements between the two radiographers, Kappa values were calculated for each individual image criteria. The Kappa values showed a spread between 0.27 and 0.44, indicating a fair agreement, according to the interpretation suggested by Landis and Koch ^(16)^. Inter- and intra-observer agreement results are shown in Table 5.

## DISCUSSION

Hand X-ray examinations require high spatial resolution and small anatomic details should be visualised both for high and low contrast resolutions. Mostly, patients are referred to hand X-ray examinations due to arthritis, arthrosis, fractures or other trauma ^(17,18)^. Thus, it is important for the patient to achieve the most precise diagnosis, which is influenced by proper image quality. When comparing image quality between different DR systems, the radiation dose used should be taken into consideration.

Even if the DAP measurements were mutually calibrated, significant variations in DAP values, for the same combinations of tube voltages and tube current-time products, between the different systems were found see Figure 1. Some of these variations were attributed to the systems´ different amount of X-ray filtration see Table 1, the primary spectral shape described by the HVL of the X-ray beam, as well as minor differences in tube voltage and tube current-time values as some of the chosen exposure parameter combinations were not available for all systems. Still some differences that cannot be explained by these factors. For instance, even if both Canon and Siemens use the same amount of X-ray filtration (2.5 mmAl), their DAP values differ significantly, especially at lower DAP values. Given possible differences in reliability and possible improper calibration for (some of) the DAP meters, one would expect errors in the DAP readings, particularly for low values. Based on statistics from the 10 exposures for each exposure parameter combination in the CDRAD experiments, the measured relative standard error of mean (SEM(DAP)/DAP) on average ranged from ~8% for the lower DAP values (≈ 0.4 Gycm^2^) to ~1% for the higher DAP values (≈ 5 Gycm^2^). This, however, cannot account for the observed differences between the systems as seen in Figure 1, because in addition to statistical variations, there are systematic differences between them. The variances thus might stem from actual differences in the anodes and/or the X-ray generators, for instance due to system construction issues and/or age. However, in that case, DAP differences would be expected to result also in differences in image quality.

When analysing and comparing the results for DAP-adjusted image quality, the α−/*a* coefficients were interpreted as offsets of IQF_inv_ and VGAs, respectively, at low DAP values, whereas the *β/b* coefficients were interpreted as image quality sensitivity to increasing DAP-values Table 3. Thus, in comparing the systems one should consider both coefficients.

Although the two regression models, logarithmic and power, respectively, gave only minor differences in quality scores for the systems, they pick up small nuances. Generally, we used the power model to discuss general (average) quality properties of the systems, and the logarithmic model to compare amongst the systems.

**Table 4:** Results of logarithmic regression modelling for i) IQF_inv_ and ii) VGAs, respectively, vs. ln(DAP)

	i) IQF_inv_ *= a + b·*ln*(*DAP*)*			ii) VGAs *= a + b·*ln*(*DAP*)*			iii) Slope ratio*: b*_VGAS_*/b*_IQF*inv*_
	*a*	*b*	*R^2^*	*a*	*b*	*R^2^*	
Agfa	3.43 (3.20–3.66)	2.40 (2.10–2.71)	0.92	3.12 (3.00–3.31)	0.91 (0.81–1.01)	0.97	0.38 (0.32–0.44)
Canon	4.70 (4.56–4.84)	1.91 (1.73–2.09)	0.96	3.70 (3.56–3.84)	0.24 (0.06–0.42)	0.38	0.13 (0.03–0.22)
Fuji	2.91 (2.85–3.06)	1.70 (1.56–1.83)	0.97	3.56 (3.41–3.71)	1.03 (0.85–1.20)	0.92	0.61 (0.49–0.72)
GE	3.07 (2.97–3.17)	1.62 (1.48–1.76)	0.96	3.46 (3.26–3.66)	0.75 (0.50–1.00)	0.76	0.46 (0.30–0.62)
Philips	2.60 (2.53–2.68)	0.90 (0.81–0.99)	0.95	2.95 (2.81–3.09)	1.14 (0.95–1.33)	0.93	1.27 (1.02–1.51)
Siemens	4.24 (4.20–4.39)	2.01 (1.90–2.13)	0.98	3.75 (3.52–3.89)	0.61 (0.45–0.78)	0.83	0.30 (0.22–0.38)
Average	3.43 (3.36–3.51)	1.79 (1.69–1.88)	0.99	3.39 (3.31–3.48)	0.79 (0.69–0.90)	0.95	0.44 (0.37–0.50)

*Note*: The right column shows the ratio b_VGAS_/b_IQFinv_. *R^2^* is the squared Pearson correlation coefficient for the fit.

**Table 5:** Inter- and intra-observer agreements per criterion (Cohen’s weighted kappa, *ĸ*).

VGA criterion	*ĸ* Inter-observer	*ĸ* Intra-observer A	*ĸ* Intra-observer B
1	0.33	0.72	0.69
2	0.39	0.83	0.78
3	0.40	0.71	0.75
4	0.27	0.93	0.32
5	0.44	0.75	0.35

### Technical image quality analysis

As shown in Figure 3a, the technical image quality metrics (IQF_inv_) showed considerable correlation with ln(DAP). Still, as can be seen from the non-overlapping confidence intervals in Table 4i, there are statistically significant differences in quality performances between the systems. In fact, at ln(DAP) = 1.1, corresponding to a DAP-value of a 50 kV/2.5 mAs exposure, typical for a PA hand X-ray examination, there is a 3.5 difference in IQF_inv_-values between Canon and Philips. While Canon, Fuji, GE and Siemens all show almost equal sensitivities in IQF_inv_ to increasing DAP (*b*-values: 1.62–2.04), Canon and Siemens in general score significantly higher than Fuji and GE across all DAP values. Although yielding low IQF_inv_ values for low DAP, Agfa has a significantly higher sensitivity (*b*-value 2.4) to increasing dose (DAP) than the others do, with the result that for high DAP values their system ranks between Canon and Siemens. Philips, on the other hand, shows the opposite trend (*b*-value 0.9) and comes out last in this technical quality comparison.

As we found no linear correlations between the quality performances (coefficients *a, b, α* and *β* in Table 4) and the system's technical specifications Table 1, these differences are hard to explain other than that the systems actually perform differently, and/or that differences in the DR system’s data processing causes the CDRAD Analyser software to perform differently. Although the power regression model also yields significant differences in the *β*-coefficients between the systems, as expected Figure 2 shows that the average value of *β* across the systems and tube voltages is 0.47 (95% CI: 0.42–0.51), indicating that IQF_inv_ scales approximately as DAP^1/2^. This result seems reasonable since the signal-to-noise ratios in a quantum-limited noisy background according to the Rose model^(5,12)^ is expected to scale as the square root of dose.

As the CDRAD phantom is designed to measure the combined effects on image quality of contrast and spatial resolution under the influence of noise, one might expect detector quality, such as detector pitch size, to influence image quality. Although the detailed results are not reported here, we found no statistically significant linear correlations between the *α*/*β* and *a/b* coefficients, respectively, and detector pitch. It is possible that the differences between the vendors found in the technical image quality analysis can be explained by differences in detector characteristics (modular transfer functions (MTF), noise power spectrum (NPS) and detective quantum efficiency (DQE), X-ray spectrum, total filtration and/or software processing of the images, which is documented by former studies ^(19–21)^.

Note from Figure 2 that there seems to be a slight negative second-order curvature in addition to the linear term, something that has also been observed by others ^(6)^, and which might be related to the fact that IQF_inv_ has a upper theoretical bound of IQF_inv,max_ = 8.89 (corresponding to ln(IQF_inv,max_) = 2.19). If one includes a second-order term in the regression model, the linear term comes out as *β* = 0.54. However, we do not explore this further here. Figure 2 might indicate some minor differences in the slopes for ln(IQF_inv_) vs. ln(DAP) between the three tube voltages (45, 50 and 55 kV). Linear regression (results not reported here) showed that these differences were not statistically significant, and may as well be attributed to the argument above regarding the upper theoretical bound of IQF_inv_.

### Visual image quality analysis

As seen from Tables 3 and 4, and illustrated in Figure 3, the VGAs also showed quite strong correlations with ln(DAP), although with some different performance ranking between the systems as compared with the technical quality results, and with an important exception for Canon. Canon performed almost independent of dose, which is difficult to explain but might be due to image processing. While Fuji, Agfa and Philips showed the highest sensitivities on VGAs from increasing ln(DAP) (regression slope a: 3.56, 3.12 and 2.95), Fuji performed better and Philips worse than Agfa across the whole DAP range (*b*-values: 0.91–1.14). On the other hand, GE and Siemens performed better at lower doses, although both showing less sensitivity to increasing dose (*b*-values: 0.61–0.75). The overall impression is that while there were quite large visual quality differences at lower doses, all systems performed, within uncertainty, quite equally at higher doses.

As both Canon and Philips used 2.5 mmAl filtration, the 4–5 orders of magnitude differences in dose sensitivity (slope *b*: 0.24 and 1.14) cannot be explained by external tube filtration alone, although the primary spectra shape are unknown. The remaining explanation candidates are differences in anode/generator properties, detector quality and/or image post-processing, but it will need further studies to decide the contributions from these factors. Although a study by Smet *et al.*^(22)^ demonstrated VGAs differences between detectors and dose levels in a neonatal phantom, this did not include image post-processing changes.

In contrast to the technical quality analysis results, the exponents from the power regression model of the VGAs results for all systems were found to be significantly lower than the Rose-theoretical value of 0.5. Even on introducing a second-order term in the line fit, with the idea that VGAs has a maximum value of 5, this still holds true. One explanation is that the visual quality impression of the images is not noise limited, but rather limited by anatomical structures ^(23)^. However, quite unlike to the technical image quality experiments (IQF_inv_) it is reassuring to observe that at ln(DAP) = 1.1, as noted above to be typical for a PA hand X-ray examination, the differences in VGAs values between the DR systems are within ~0.6 units, and which can be assumed to be within statistical uncertainty.

### Correlations between visual and technical image quality

A clear correlation between visual and technical image quality figures of merit for each system was found. However the relationships between VGAs and IQF_inv_ values vs. ln(DAP) amongst them are quite different Figure 3. The slope ratios between the visual and technical logarithmic regressions (*b*_VGAS_*/b*IQF_inv_) range from 0.13 (Canon) to 1.27 (Philips), which both, as can be seen from their non-overlapping confidence intervals in Table 4iii, are significantly different from the others in this respect. Agfa, Fuji, GE and Siemens, within uncertainties, are equal, although Fuji has a significantly higher slope ratio than Siemens.

Several groups have studied the possibility of using CDRAD phantom results (IQF_inv_) as an indicative measure of visual image quality in clinical imaging optimisation. Al-Murshedi *et al.*^(8)^, used images of a Lungman chest phantom for the visual quality assessment and a Konica Minolta DR system, and found strong positive correlation (*r* = 0.91; *p* < 0.001) between visual quality and IQF_inv_. On the other hand, Konst *et al.*^(6)^, used a Canon CXDI-40EG detector, and concluded, due to too significant uncertainties in the obtained IQF_inv_ values, that ‘CDRAD may not be an optimal phantom to differentiate between images acquired at different dose levels’. Mourik *et al.*^(24)^ when using a CDRAD phantom to analyse chest-imaging quality between seven different wireless detectors reached the same conclusion. They compared IQF_inv_ values for clinical protocols (0.5–0.9 mAs/117–120 kVp) from various hospitals and a reference protocol, where all technical parameters were aligned. Their study showed large uncertainties in IQF_inv_, and no clear correlation between IQF_inv_ and dose for most of the systems. Although this may seem to oppose our results, their experiments used relatively low doses, producing larger relative uncertainties.

Our study supports the result that phantom measurements may be used as indicative measures of visual quality. Still, one should be cautious to compare visual quality between systems based on CDRAD phantom measurements. Yalcin *et al.* recently published similar results, although for a different set of DR systems and using a lung/chest phantom for the visual experiments ^(25)^. As noted previously, there is a possibility that differences in the DR system’s image processing might cause the CDRAD Analyser software to perform differently. This should be further investigated.

### Limitations

The standard way to assess technical image quality of the DR detectors is to calculate DQE based on measurements of MTF and NPS. Such measurements cannot easily be performed since raw image data are not available to the users when the detectors are integrated into clinical systems. In this study, it was not possible to measure technical metrics of all imaging detectors; therefore, IQF_inv_ and perceived image quality (VGA) were assessed.

As only two readers participated, it seems reasonable to assume that evaluation of clinical image quality is biased by the image impression generated by the various systems, which in large part is influenced by image processing algorithms and not assessed in this study. Our clinical experience is that readers tend to prefer the image appearance that they are familiar with from their daily routine. The subjective image quality VGA-scores may reflect this fact, and it would be interesting to include readers using all of the evaluated systems. In other studies, where all images were from the same system, strong correlation between technical and subjective image quality was found.

## CONCLUSIONS

This study demonstrates the differences between the DR systems included. When compensating for variations in dose it showed that *a* in the technical experiments/visual experiments and between them, one particular DR system could yield the best quality scores at some exposure parameter ranges and the worst in others.

The technical image quality results showed that Canon had the highest IQF_inv_ values over all dose levels, just followed by Siemens. Philips had the lowest IQF_inv_ score across all dose levels included, and in addition the lowest dose response (regression slope). Agfa scored the lowest IQF_inv_ for lower doses but had the best responses of image quality to dose.

On the other hand, the visual image quality results showed that Siemens and Fuji had superior VGAs for most dose levels, while Philips and Agfa did show the lowest VGAs. The best dose responses were found for Philips and Fuji and the lowest dose responses were observed for Canon.

For a typical clinical dose for DR hand examinations (50 kV, 2.5 mAs, corresponding to approximately ln(DAP) = 1.1, the results for technical image quality showed quite large differences between the systems, with Canon scoring the highest and Philips the lowest, while for visual image quality, within statistical uncertainty, the systems scored equally. Thus, our experiments also showed that using a CDRAD phantom as a method to mimic and compare visual image quality between different DR systems can be quite dangerous.

Further studies are warranted to explore different anatomies and implement these results on patients.

## DISCLOSURES

None.
